# The role of the six factors model of athletic mental energy in mediating athletes’ well-being in competitive sports

**DOI:** 10.1038/s41598-024-53065-5

**Published:** 2024-02-05

**Authors:** Amisha Singh, Mandeep Kaur Arora, Bahniman Boruah

**Affiliations:** 1 Department of Psychology, University of Delhi, New Delhi, India; 2 Department of Psychology, Kamala Nehru College, University of Delhi, New Delhi, 110007 India

**Keywords:** Psychology, Human behaviour

## Abstract

In the realm of high-performance sports, athletes often prioritize success at the expense of their well-being. Consequently, sports psychology researchers are now focusing on creating psychological profiles for athletes that can forecast their performance while safeguarding their overall well-being. A recent development in this field is the concept of athletic mental energy (AME), which has been associated with both sporting success and positive emotions. Therefore, the aim of this study was to explore if AME in athletes can mediate this directly observed relationship between performance and psychological well-being. For stronger predictive validity these relationships were examined across two studies with each involving distinct sets of participants engaged in various sports disciplines, including football, cricket, basketball, archery, and more. The self-report measures of sports performance, athletic mental energy (AME), and psychological well-being (PWB) were administered post-competition on the local, regional, state, national, international, and professional level athletes of age 18 and above. Our study found that both, the affective and cognitive components of AME mediated the athletes’ performance and psychological well–being relationship. Interestingly, the study found no significant gender differences in AME and PWB scores. While family structures didn’t yield significant variations in AME scores, there were some descriptive distinctions in PWB scores across different family structures. Our research offers preliminary evidence suggesting that AME can play a pivotal role in preserving athletes’ psychological well-being following competitive events.

## Introduction

Engagement in sports has a profound impact on the physical, psychological, and social well-being of athletes, as demonstrated by various studies indicating that sports training leads to improvements in athletes’ cardiovascular health, neuromuscular function, and respiratory capacity, along with other physiological benefits such as enhanced metabolism, better sleep, and a strengthened immune system^1–3^. Beyond the physical advantages, sports participation also nurtures essential psychological skills like self-esteem, competence, resilience, and motivation, all of which can positively influence various aspects of an athlete’s life^1^. Furthermore, organized sports activities offer a platform for athletes to communicate, build relationships, collaborate, and cultivate a sense of belonging^4^. However, it’s important to acknowledge that an individual’s engagement in competitive sports is not without its challenges and stressors^1,5^. Athletes confront a range of stressors stemming from their training and competitions, including sport-specific challenges (e.g., the pressure to win, injuries, and team selection), general life issues (e.g., family relationships and economic conditions), and organizational aspects (e.g., team selection, accommodations, and travel support)^6–11^. These challenges can lead to both physical and psychological problems for athletes. Physically, stress can increase the risk of cardiovascular disease, hyperglycemia, stomach and intestinal ulcers, and asthma^12–15^. Psychologically, athletes may experience decreased well-being, deteriorating sports performance, mood disorders, eating disorders, depression, anxiety, overtraining, attention-deficit/hyperactivity disorder, burnout, and increased episodes of depression^16–19^. Additionally, the stress resulting from past unsatisfactory performance can manifest as pain, discomfort, and performance anxiety, all of which can significantly affect an athlete’s well-being and performance efficiency^20–22^.

Athletes need psychological skills to tackle these challenges by managing emotions, handling stressors, and achieving their goals^23,24^. Training in these skills not only fosters effective coping strategies but also yields psychological benefits that boost their performance and overall well-being in competitive sports, making them crucial for success ^25,26^.

Over time, the field of sports psychology has shifted its focus from solely improving athletic performance to considering athlete well-being as an integral part of performance^27^. Well-being is a multidimensional phenomenon that encompasses cognitive and affective dimensions resulting from an individual’s assessment of various aspects of life^28^. The close connection between performance and psychological well-being in sports has prompted researchers to investigate the underlying factors influencing the performance-well-being relationship, enabling practitioners to develop programs and interventions aimed at preventing ill-being resulting from underperformance or high-performance expectations.

One emerging concept of particular interest in sports is "athletic mental energy (AME)," which may play a vital role in athletes’ performance and psychological well-being. Generally, mental energy refers to an individual’s capacity to concentrate on a task and block out distractions^29^. Notably, historical figures like Newton, Galileo, Archimedes, and Einstein possessed strong mental energy, which enabled them to accomplish remarkable feats^30^. The International Life Science Institute (ILSI) suggests that mental energy relates to an individual’s belief in their ability to accomplish a given task. Lu et al.^79^ expanded on the ILSI concept of mental energy and defined AME as a multifaceted construct specific to sports, characterized by an athlete’s perceived state of energy, comprising cognitive components such as confidence, concentration, motivation, and mood. These components have been frequently observed in flow research, where Olympic athletes with high AME report an optimal level of activation necessary for peak performance, success, and experiences of flow^31–39^. For instance, athletes with higher confidence and concentration tend to fear failure less, perform better, report better performance, and have more frequent experiences of flow during optimal performance^40–44^. Motivation is also recognized as a vital component of AME, with numerous studies in sports psychology emphasizing its relevance for peak performance. The emotional components of AME, including vigor, calmness, and tirelessness, are positively correlated with athletic performance, well-being, and a positive mental state^38,45–47^. High AME has been associated with lower athletic burnout and stress, moderating the negative effects of stress and burnout in athletes^48^. For example, research suggests that Olympic medalists in rowing with high vigor experience lower levels of depression and fatigue compared to their counterparts^49,50^. In alignment with the principles of positive psychology, AME encompasses positive elements that can help athletes manage stress resulting from perceived performance and safeguard their well-being. Therefore, it is reasonable to assume that athletes with high AME are more likely to deliver exceptional sports performance, exhibit greater engagement and consistency in training and competitions, and experience higher levels of well-being compared to athletes with lower AME levels. However, further research is necessary to validate these assumptions.

Researchers are increasingly interested in developing athletes’ mental and emotional profiles to predict both their performance and well-being. The predictive validity of such profiles holds theoretical and practical implications for future research and intervention development. To our knowledge, this study represents the first attempt to examine the influence of AME on the relationship between athletes’ performance and psychological well-being post-competition. Additionally, our study draws support from various established models and concepts in psychology, including Morgan’s iceberg profile, Hanin’s Individual Zone of Optimal Functioning (IZOF), the Profile of Mood States (POMS) by McNair, Lorr, and Droppleman, psychological skills by Mahoney et al.^53^, performance strategies by Thomas et al.^54^, mental skills by Durand-Bush et al.^55^, mental toughness by Jones et al.^56^, and resilience by Fletcher and Sarkar^35,51–56^. These models have consistently demonstrated the relationship between performance and psychological state in athletes, further reinforcing the significance of our research in exploring the mediating role of AME in athletes’ performance and psychological well-being.

## The present study

Athletes experience significant pressure to excel in competitive sports, impacting their psychological well-being. Poor performance can exacerbate stress and anxiety, harming mental health. Nonetheless, recent research suggests that harnessing athletic mental energy (AME) can help preserve athletes’ well-being. Therefore, through our research we aimed to investigate the connection between sports performance and psychological well-being, using athletic mental energy (AME) as a mediator. The hypothesis was that AME would play a mediating role in the relationship between performance and psychological well-being. Two cross-sectional studies with quantitative research designs were conducted with different groups of participants. Study 1 aimed to explore the mediating effects of AME on the relationship between an athlete’s performance and psychological well-being, while Study 2 sought to replicate the findings of Study 1 and further confirm the role of AME as a mediator in the relationship between athletes’ performance and psychological well-being.

### Study 1

#### Purpose

To examine the mediating effects of athletic mental energy on the relationship between athletes’ performance and psychological well-being.

### Methods

#### Participants

For study 1 participants were 50 athletes (males = 50%) with an average age of 20.34 years (SD =  ± 1.86) from 10 states of India. At the time of the data collection, 70% of participants were regularly engaged in competitive sports from 15 different individual and team sports, including archery (6), athletics (7), bodybuilding (1), baseball (1), boxing (1), kho-kho (1), rock-climbing (1), table tennis (1), sprinting (1), taekwondo (1), cricket (3), hockey (3), football (12), kabaddi (4), and volleyball (7). The sample was representative of diverse competitive levels, with 58% of participants being national and international athletes.

The average hours of engagement in sports training per week were 23.04 h (SD = 10.68), furthermore, on average the athletes were active in sports for 3.86 (SD = 1.94) years. Lastly, some exclusion criteria were pre-defined across both studies including the exclusion of winter and traditional sports, players competing in parasport, and participants who had not participated in any competitive encounters for the last month (one week for participants in study 2) due to injury, illness, or non-selection.

#### Measurements and procedures

The study adhered to the American Psychological Association’s (APA) Code of Ethics throughout its entirety. The Departmental Research Council (DRC) approved the study before initiation. Coaches, physical education instructors, and sports clubs were informed of the research’s purpose via email and phone calls by the lead author. Subsequent agreements included details on contacting players, assessment duration, location, and other requirements. During appointments with participants, the study’s purpose, nature, design, and ethical rights were explained, including informed consent, debriefing, anonymity, data safety, confidentiality, and the right to withdraw. Participants received a hard copy of a multi-section questionnaire which was built through the approval of the experts including 2 academicians, 2 counselors, and 1 psychometrician for the sequence and presentation of the Questionnaire. Section A collected demographic and sports profiles (e.g., gender, location, age, family structure, sport category, types of sports, highest competition participated in, and years of athletic experience). Section B included psychological scales: Performance Satisfaction (PS), Psychological Well-being (PWB), and Athletic Mental Energy Scale (AMES). To mitigate social desirability bias, participants were informed that the study aimed to understand their life experiences as sports performers, with no right or wrong answers. Mental energy, as a state construct, is best measured close to competition to reflect the exact mental and emotional state^38,57–59^. However, participants completed the scales within one month after the competition due to tight schedules and depleted energy, mood, and motivation during competitions. They were asked to reflect on their sporting experiences from the past month when responding to items.

#### Demographic questionnaire

In section A, the demographic profile questionnaire contains participants’ information including gender, location, age, and family structure while the sport profile questionnaire contains participants’ information related to sports including sport category, types of sports, highest competition participated in, and years of athletic experiences, etc.

##### Psychological well-being (PWB)

Psychological Well-being (PWB) is an 18-item scale by Ryff and Keyes (1995), assessing six components: autonomy, environmental mastery, purpose in life, personal growth, positive relations with others, and self-acceptance. Participants rated their agreement with each statement on a 7-point scale (1 = strongly agree; 7 = strongly disagree). Cronbach’s α for the total PWB score was 0.78^60–62^. For the final analysis, we used the total score of PWB.

##### Athletic mental energy scale (AMES).

The athletic mental energy scale (AMES), by Lu et al.^79^, comprises 18 items measuring athletes’ perception of their energy state, including vigor, confidence, motivation, tirelessness, concentration, and calm. Participants rated their feelings on a 6-point Likert scale (1 = not at all, 6 = completely so). Cronbach’s α for the total AMES score was 0.92^48,63^. We used the total score of AMES for the main analysis in Study 1 and scores on each subscale in Study 2.

##### Subjective performance (SP)

Subjective Performance scale (SP) by Brown (2017) was used to assess participants’ satisfaction with past competitive performances over a month (or a week in Study 2) using an 11-point rating scale (0 = totally dissatisfied, 10 = totally satisfied; cf. Pensgaard and Duda, 2003). This approach allows for comparisons among athletes across different sports, offers sensitivity in performance assessment, and minimizes environmental influences (for example, opponent’s skill level)^64–68^.

#### Statistical analyses

Statistical analysis has been performed with SPSS (Statistical Package for Social Sciences) Version 23. None of the participants were identified as univariate outliers through the inspection by a boxplot. All the participants were kept for analysis. Furthermore, the scores of AMES, PWB, and SP were normally distributed through the assessment of Q-Q plots and the Shapiro–Wilk test (W 50) = 0.84, p = 0.07). Furthermore, we used mediation analysis using Hayes Process v4.1 macro in examining the mediating effects of the AMES on the performance and psychological well-being relationship. The significance level across both studies was set at p < 0.05.

### Results

The reliability of a measure in any research study is the measure of internal consistency and is accepted if the Alpha (α) value of the latent construct is greater than 0.70^69^. Internal consistency reliability for the current study was assessed using Cronbach’s Alpha. The results revealed that the AMES with 18 items (α = 0.92) and the PWB Scale with 18 items (α = 0.78) were found reliable for Indian athletes (n = 50).

The sample of athletes (N = 50) consisted of 25 (50%) females with the highest percentage of athletes from Delhi i.e., 25 (50%), followed by Haryana and Uttar Pradesh i.e., 7 (14%). The remaining athletes were from Bihar, Madhya Pradesh, Manipur, and Mumbai 1 (2% each), 4% from Himachal Pradesh and Jharkhand (2 each), and 3 (6%) from Rajasthan. Furthermore, 17 (34%) athletes were living in joint families, 19 (38%) in nuclear families, and 14 (28%) in single-parent families. For sport profile, 19 (38%) sample of athletes belonged to the individual and 31 (62%) belonged to the team sport category, 13 (26%) Junior category and 37 (74%) belonged to the senior category. The sport category of the participants was 01 each for baseball, bodybuilding, boxing, kho-kho, rock climbing, sprinting, table tennis, taekwondo (2% each), 03 each in Cricket and hockey (6% each), 04 in kabaddi (8%), 06 in Archery (12%), 07 in athletics (14%), and 12 in Football (24%). A maximum number of athletes were engaged in a regular training session 35 (70%). Lastly, in the current sample of athletes, 4(8%) were professionals, 5(10%) were international-level athletes, 24 (48%) were national-level athletes, 14 (28%) were state-level athletes, and 3 (6%) were regional and local-level athletes.

We found an overall mean score of 4.23 (SD = 0.97) for AME, indicating an overall moderate AME experience amongst athletes. Motivation ($\overline{{\text{X}}}$ = 4.62; SD = 1.04) and Concentration ($\overline{{\text{X}}}$ = 4.41; SD = 1.05) had the highest mean values, demonstrating that players feel motivated and persist in high concentration levels during training or competition. Furthermore, an overall mean score of PWB ($\overline{{\text{X}}}$ = 4.81; SD = 0.74) shows an overall moderate PWB experience amongst athletes. Personal growth ($\overline{{\text{X}}}$ = 5.28; SD = 1.17) and Self-acceptance ($\overline{{\text{X}}}$ = 5.12; SD = 0.95) in PWB had the highest mean values, demonstrating that the players generally have a sense of growth, are open to new experiences, and have a positive attitude towards themselves through the acknowledgment and acceptance of all the aspects in life. Lastly, the descriptive statistics for SP reveal overall moderate (M = 6.3; SD = 1.66) SP experiences amongst athletes.

Also, the data was collected from athletes from three different family structures i.e., joint (n = 15), nuclear (n = 16), and the single-parent family (n = 19), and the results from descriptive statistics reveal an overall mean score of 83.66 (SD = 21.97) for the psychological well-being of athletes living in a joint family, 83.50 (SD = 20.01) for the nuclear family, and 83.62 (SD = 18.93) for a single-parent family.

**Figure 1:**
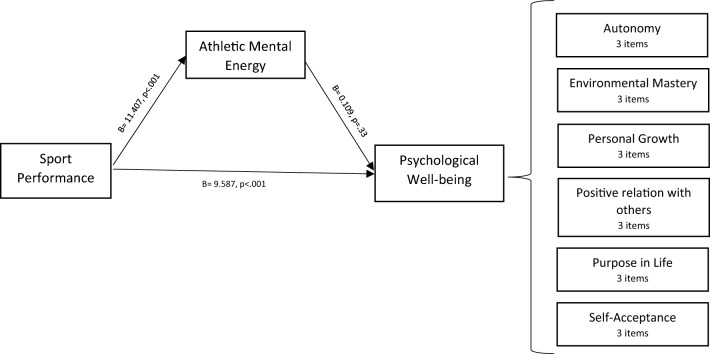
Mediation analysis representing the relationship between Sport performance and psychological well-being with athletic mental energy as a mediator.

Lastly, the study assessed the mediating role of AME on the post-competitive relationship between subjective performance and psychological well-being. The results demonstrated a non-significant indirect effect of the impact of subjective performance on psychological well-being (b = 1.24, t = 7.0). However, the direct effect of subjective performance on PWB in the presence of a mediator was found significant (b = 9.58, p < 0.001). Hence, athletic mental energy did not mediate the relationship between subjective performance and PWB. To save space, we show only the figure of the mediating effect of AME on the athletes’ performance-psychological well-being relationship (refer to Fig. 1). The mediation analysis summary is presented in Table 1.

**Table 1:** Mediation analysis representing the relationship between Sport performance and psychological well-being with athletic mental energy as a mediator.

Relationship	Total effect	Direct effect	Indirect effect	Confidence interval		t-statistics	Conclusion
				Lower bound	Upper bound		
SP—> AME—> PWB	10.8312 (p = 0.000)	9.5872 (p = 0.000)	1.2440	−1.3081	3.5195	7.0412	Insignificant mediation

*SP* subjective performance, *AME* athletic mental energy, *PWB* psychological well-being.

### Discussion for study 1

Our study extended Lu et al.^79^ work on AME by examining its mediating effects on the relationship between athletes’ performance and psychological well-being post-competition. The results of the descriptive statistics on the mean score of the psychological well-being of athletes from a joint family, nuclear family, and single-parent family indicate some group differences. However, the significance of this group difference is yet to be tested. Furthermore, the results from the mediation analysis revealed that athletic mental energy did not mediate the relationship between sport performance and PWB. Nonetheless, the first study provides preliminary evidence of the direct effect of athletes’ performance on PWB. Furthermore, the results of our study could be explained either due to the small sample size or due to the long gap time interval between the participants’ involvement in sports competition and the conduction of the test which ultimately fails to record the actual state of athletes’ mental energy in relation to their competitive performance. Therefore, for the second study, a greater number of participants and a lesser time frame for test administration shall be kept for the participants to reflect their exact psychological state relating to their competitive encounter.

### Study 2

#### Purpose

The aim of our second was to recreate Study 1 and further confirm the mediating effects of AME on the athletes’ performance and psychological well-being relationship.

### Methods

#### Participants

For study 2 participants were 100 athletes (males = 50%) with an average age of 23.11 years (SD =  ± 2.32) from 14 states in India. At the time of the data collection, 78% of athletes were regularly participating in training seasons. Also, the participants were from 15 different types of individual and team sports including aerobics (3), athletics (15), archery (1), badminton (4), basketball (11), baseball (1), cricket (3), football (20), handball (2), hockey (4), kho-kho (8), roller skating (1), sprinting (2), table tennis (2), and volleyball (23). 40% of participants were national level players followed by 5% professional and international level players. The average hours of engagement of athletes in sports training per week were 15.77 h (SD = 9.54) and the number of years athletes were active in sports on average was 4.35 years (SD = 1.14). The exclusion criteria were kept the same as in Study 1.

#### Measurements and procedures

Study 2 followed a procedure consistent with Study 1, maintaining data collection methods, assessment measures, and statistical analyses. The Cronbach’s α for Psychological Well-being (PWB) and athletic mental energy scale (AMES) in Study 2 were 0.74 and 0.93, indicating satisfactory internal consistency. Upon securing informed consent, participants received a multi-section questionnaire. To align with the need to capture participants’ accurate psychological states, AME assessments were conducted within one week of the competitive encounters^25,45,58,59^. Participants who agreed to this timeline were included in the study, while others were excluded. Participants were instructed to reflect on their experiences from competitive sports encounters in the past week when responding to the questionnaire items.

#### Statistical analyses

In Study 2, data analysis paralleled the procedures in Study 1. All data were initially screened to ensure they met the prerequisites for t-tests and ANOVA. Univariate outliers were absent based on boxplot examination, resulting in the retention of all participants for analysis. Normality checks for psychological well-being, athletic mental energy, and subjective performance were performed using skewness and kurtosis values within acceptable limits (skewness between ± 2 (George and Mallery, 2020), and kurtosis from a range of − 10 to + 10 (Collier, 2020), alongside the Kolmogorov–Smirnov test confirming a normal distribution (W (100) = 0.053, p = 0.20). This mediation analysis was executed in two phases, initially examining the total AME score in Phase 1 and subsequently assessing the scores for the six AME factors.

## Results

As assessed through Cronbach’s Alpha, the scales, AMES with 18 items (α = 0.93) and the PWB Scale with 18 items (α = 0.74) were found reliable for Indian athletes (n = 100).

The participants (N = 100) consisted of 50 (50%) females and 50 (50%) male athletes with the highest percentag e of athletes from Delhi i.e., 53%, followed by Haryana (18%) and Uttar Pradesh (17%). The remaining athletes were from Rajasthan (2%), followed by Arunachal Pradesh, Bhopal, Bihar, Chhattisgarh, Jammu & Kashmir, Jharkhand, Manipur, Mumbai, Punjab, and Uttarakhand (10%). Furthermore, 34% of athletes belonged to joint families with an equal percentage of athletes from nuclear families, and 32% of athletes were from single-parent families. For the sport profile, 22% of athletes belonged to the individual sport category and 78% belonged to the team sport category in which 22% Junior category, and 78% belonged to the senior category. The highest percentage of athletes were from Volleyball (23%) followed by football (20%), athletics (15%), basketball (11%), kho-kho (8%), and remaining other sport categories (23%). A maximum percentage of athletes were engaged in a regular training session (78%). Lastly, in the current sample of athletes, 3% were professionals, 2% were international-level athletes, 40% were national-level athletes, 18% were state-level athletes, and 37% were regional and local-level athletes.

The results from descriptive statistics for AME reveal overall moderate AME experiences amongst athletes with a mean score of 4.12 (SD = 0.95). Vigor ($\overline{{\text{X}}}$ = 4.17; SD = 1.13) and Motivation ($\overline{{\text{X}}}$ = 4.61; SD = 1.10) had the highest mean value, demonstrating that the athletes felt energetic and enthusiastic about sports and generally felt motivated towards training and competition Similarly, descriptive statistics for PWB reveal overall moderate PWB experiences amongst athletes (M = 4.79; SD = 0.65). Similar to the study1, Personal growth ($\overline{{\text{X}}}$ = 5.32; SD = 1.08) and self-acceptance ($\overline{{\text{X}}}$ = 5.06; SD = 0.82) had the highest mean value. Also, the descriptive statistics for SP show overall moderate SP experiences amongst athletes (M = 6.22; SD = 1.64).

Similar to study 1, the data was collected from athletes belonging to three different family structures including the joint family (n = 34), the nuclear family (n = 34), the single-parent family (n = 32), and, the overall descriptive statistics show the difference in the mean scores on the psychological well-being of athletes belonging to the joint family ($\overline{{\text{X}}}$ = 83.85, SD = 17.84), the nuclear family ($\overline{{\text{X}}}$ = 77.85, SD = 14.27), and the single-parent family ($\overline{{\text{X}}}$ = 86.71, SD = 21.52).

Furthermore, an independent sample t-test was conducted to compare the Athletic Mental Energy and Psychological well-being of Male and Female athletes. The mean difference for PWB (− 0.18) and AME (− 0.11) was calculated using Cohen’s d and a small effect size was found for male and female participants indicating negligible difference between both the groups. No significant differences (t (98) =  − 0.56, p = 0.57) were found in the scores of Males ($\overline{{\text{X}}}$ = 71.26, SD = 15.40) and Females ($\overline{{\text{X}}}$ = 73.16, SD = 18.41) for Athletic Mental Energy. The magnitude of the differences in the means (mean difference = − 0.11, 95% CI: − 0.51 to 0.28) was very small. Similarly, for psychological well-being, there were no significant differences (t (98) = -0.92, p = 0.35) in scores for Males ($\overline{{\text{X}}}$ = 81.04, SD = 17.17) and Females ($\overline{{\text{X}}}$ = 84.42, SD = 19.28) and the differences in the magnitude of the means (mean difference = − 0.18, 95% CI: − 0.58 to 0.21) was very less.

Additionally, we used one-way ANOVA to test if the AME of athletes differs across different family structures. Participants were divided into 3 groups (Group 1: Joint family; Group 2: Nuclear family; Group 3: Single-parent family). The ANOVA results suggest that the Athletic Mental Energy does not differ significantly (F2, 97 = 1.44, p > 0.005).

**Figure 2:**
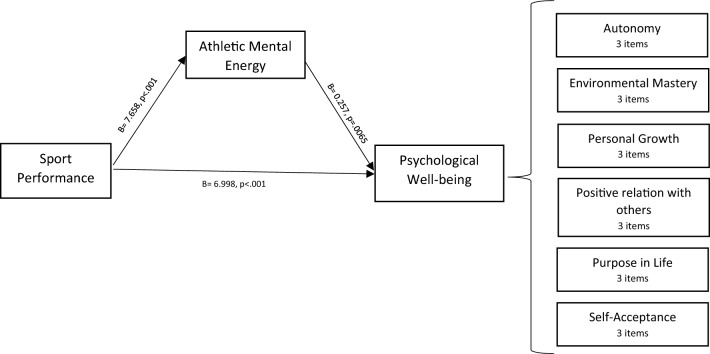
Mediation analysis represents the relation between performance and psychological well-being with athletic mental energy as a mediator.

Lastly, the study assessed the mediating role of AME on the relationship between SP and PWB in phase 1. The results revealed a significant indirect effect of the influence of SP on PWB (b = 1.97, t = 2.33), supporting our study hypothesis. In addition, the direct effect of SP on PWB in the presence of a mediator was also found significant (b = 6.99, p < 0.001). Hence, a total score of AME mediated the relationship between SP and PWB (refer to Fig. 2.). The summary of the mediation analysis is presented in Table 2.

**Table 2:** Mediation analysis represents the relation between performance and psychological well-being with athletic mental energy as a mediator.

Relationship	Total effect	Direct effect	Indirect effect	Confidence Interval		t-statistics	Conclusion
				Lower bound	Upper bound		
SP- > AME—> PWB	8.972 (p = 0.000)	6.998 (p = 0.000)	1.973	0.442	3.712	2.333	Partial mediation

*SP* subjective performance, *AME* athletic mental energy, *PWB* psychological well-being.

### Study 2 part B

It was further hypothesized that each of the six factors of AME i.e., vigor, confidence, motivation, concentration, tirelessness, and calm mediate the relationship between performance and PWB.

**Figure 3:**
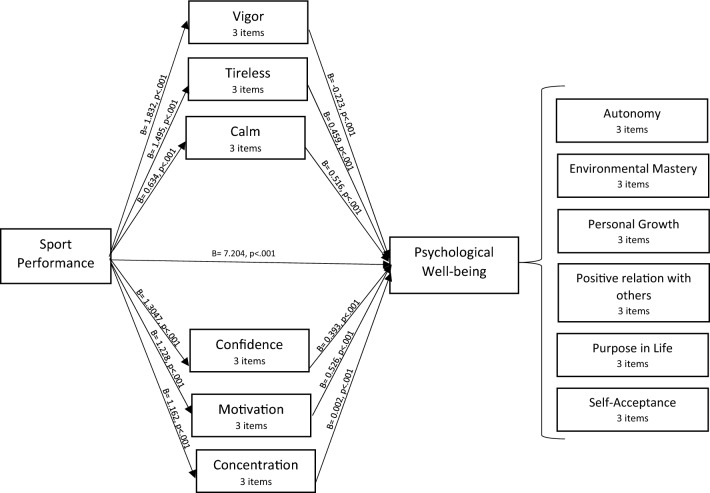
Mediation analysis representing the relationship between performance and psychological well-being with affective (vigor, tirelessness, and calm) and cognitive components (confidence, motivation, concentration) of Athletic Mental Energy as mediators.

For this, the study assessed the mediating role of each of the subdimensions of AME including vigor, confidence, motivation, concentration, tireless, and calm on the relationship between players’ performance and PWB. The results unveiled a significant indirect impact of subjective performance on psychological well-being through vigor (b = − 0.41, t = 3.41). The minus sign indicates competitive partial mediation asserting that a portion of the effect of players’ performance on players’ PWB is mediated through vigor, whereas subjective performance still explains a portion of psychological well-being that is independent of vigor. Additionally, the results revealed a significant indirect impact of subjective performance on psychological well-being through confidence (b = 0.51, t = 2.33), motivation (b = 0.64, t = 2.77), concentration (b = 0.002, t = 2.22), tireless (b = 0.68, t = 1.96), and calm (b = 0.32, t = 2.16) indicating complementary partial mediation supporting our hypothesis. Moreover, the direct effect of player’s performance on psychological well-being in the presence of the mediators was also found significant (b = 7.20, p < 0.001). Hence, all six factors of AMES partially mediated the relationship between SP and PWB (refer to Fig. 3). The summary of the mediation analysis is presented in Table 3.

**Table 3:** Mediation analysis of 6 factors of AMES.

Relationship	Total effect	Direct effect	Indirect effect	Confidence interval		t-statistics	Conclusion
				Lower bound	Upper bound		
SP→VIG→ PWB	8.972 (p = 0.000)	7.204 (p = 0.000)	− 0.410	1.766	1.539	3.416	Partial mediation
SP→CONF→PWB	8.972 (p = 0.000)	7.204 (p = 0.000)	0.513	0.6434	1.870	2.331	Partial mediation
SP→MOT→PWB	8.972 (p = 0.000)	7.204 (p = 0.000)	0.647	0.310	1.687	2.776	Partial mediation
SP→CON→PWB	8.972 (p = 0.000)	7.204 (p = 0.000)	0.002	0.888	1.139	2.222	Partial mediation
SP→TIR→PWB	8.972 (p = 0.000)	7.204 (p = 0.000)	0.687	0.937	2.806	1.962	Partial mediation
SP→CAL→PWB	8.972 (p = 0.000)	7.204 (p = 0.000)	0.327	0.158	0.936	2.165	Partial mediation

*SP* subjective performance, *VIG* vigor, *CONF* confidence, *MOT* motivation, CON concentration, *TIR* Tireless, *CAL* calm, *PWB* psychological well-being.

#### Discussion for study 2

The aim of our second study was to replicate Study 1 and uncover further evidence on the mediating effects of AME on the athletes’ performance-wellbeing relationship. With a different set of participants, a larger sample size, and minimizing the timings of test conduction from the competition, we found that the total score of AME along with the six factors of AME mediated the sport performance and psychological well-being relationship. Thus, Study 2 provided initial evidence of the original aim of the study. The theoretical and practical implications, future directions, and research limitations are thoroughly interpreted and discussed in the section below.

### Ethics statement

To fairly meet the ethical criteria, APA ethical guidelines were followed while conducting the study which included taking informed consent, ensuring participants’ right to withdraw throughout the study, ensuring anonymity, and data safety. The research has been approved by the Departmental Research Committee of the Department of Psychology, University of Delhi, India.

### Consent to participate

All the participants signed the consent form before taking part in the research.

## General discussion

Considering mental energy as an important factor in athletes’ well-being, we extended Lu et al.^79^ work on AME and investigated its mediating effects on the relationship between sport players’ performance and well-being. The results revealed a significant indirect effect of performance on psychological well-being through AME. Hence, AME mediated the relationship between performance and PWB. Moreover, all three affective (i.e., vigor, tireless, and calm) and cognitive components (i.e., confidence, concentration, and motivation) of the AME played significant roles in mediation.

The first emotional component i.e., Vigor is considered an individual’s feeling with elevated arousal^49,50^. Hence, athletes with heightened vigor would maximize their efforts in strengthening their performance with enthusiasm irrespective of their performance-related setbacks. Similarly, we found that athletes with high levels of vigor were better able to deal with performance-related setbacks and scored higher on well-being dimensions. Research also indicates that while dealing with challenges and obstacles, individuals can get back to equilibrium psychologically, physically, and socially by exerting more effort in addressing the problem and overcoming the obstacles^70^. Thus, it is highly likely that athletes high in vigor put more effort into coping with the existing performance-related pressures and stressors in competitive sports, and thus, are better able to preserve their well-being as compared to their counterparts. In support, Morgan^51^, (1980) found that Olympic athletes who scored high on vigor were more successful and had lower depression, fatigue, anxiety, anger, and confusion as compared to athletes who scored low on vigor^51^. Furthermore, similar to Lu et al.^79^ conception of vigor and tirelessness, we found that the players’ levels of well-being were impacted by their performance through tirelessness. Chuang, Lu, Gill, and Fang^38^ also contended that athletes who performed extremely well during sport competitions reported experiencing a heightened sense of control, energy, and emotional equilibrium^38,46^. Conversely, athletes who feel dissatisfied with their performance in sports are at higher risk of experiencing a lower sense of energy and disequilibrium, thus compromising their sense of well-being. Therefore, preserving athletes’ energy levels post-competition through techniques involving physiological and psychological recovery becomes an important aspect of maintaining their improved sense of well-being as well as preparing them for the next competition^46^. Furthermore, our findings on the mediating effects of calm in a performance-well-being relationship are very insightful in sport psychology research as Loehr^46^ contended that elite athletes should experience a state of calm by being mentally and physically relaxed and should not have fear of failure even if they encounter highly demanding situations during a competitive encounter^53^. Since athletes’ past experiences of unsatisfactory performance can create some level of future performance anxiety in athletes leading to heightened arousal and distraction, it is likely that athletes who have the skill to remain calm can ease their performance anxiety, and are better able to cope with their fear of failure, and maintain their sense of physical and mental well-being^13,23,31,71–73^. Furthermore, athletes with such emotional stability have reported high levels of resilience in them^35,74^. Thus, there is a higher possibility that athletes who tend to remain calm are better able to recover quickly from performance-related setbacks. In support, the calm factor of AME is frequently found in flow research wherein athletes who report sensations of calmness, relaxation, and effortlessness while engaging in any activity frequently experience flow that helps them to fully immerse themselves in preparing for the next competition without getting distracted from the thoughts related to fear of failure or experiencing performance-related anxiety^32,47^.

Just like the affective components of AME, the cognitive components including confidence, concentration, and motivation played an equally important role in mediating the athletes’ performance-wellbeing relationship post-competition. The confidence factor of AME indicates an athlete’s belief in one’s ability to accomplish a task^35,75^. The sport–confidence model also suggests that a high level of confidence in athletes can trigger adaptive emotions and larger efforts to deal with adversities, challenges, and sport-specific stressors including in-match failure and performance demands. These adaptive emotions help athletes to manage future performance anxiety and maintain a sense of well-being^35,36,68,75^. In addition to this, concentration played an important role in mediating the relationship between performance and well-being. Concentration refers to an individual’s mental ability to block unwanted distractions while focusing one’s attention on achieving a given task^33,44,75^. Athletes with strong concentration excel in competitions by enhancing focus, blocking past performance-related thoughts, reducing stress, and maintaining well-being^37,77,78^. Conversely, low concentration in athletes leads to impaired focus during stress, hindering working memory efficiency^63,79^. Such athletes struggle to concentrate, easily getting distracted by thoughts of past failures, ultimately causing increased anxiety, stress, and decreased well-being. Thus, our finding of the positive impact of the concentration on performance and psychological well-being relationship is consistent with previous research. Lastly, the mediating effect of motivation on the post-competitive performance- psychological well-being relationship has also been evident in our study. Motivation is the intensity of goal-directed behavior^42,80,81^. Generally, highly motivated individuals possess higher persistence to achieve their goals. Therefore, an athlete with higher motivation to perform well would persist longer in training to perform his best even when facing adversity^70,82^. Thus, sport players with high levels of motivation will exert more effort to handle the competing demands during sports competitions regardless of their past record of unsatisfactory performance. Thus, preserving their mental well-being. Hence, all six factors of AMES played important roles in mediating the relationship between athletes’ performance and psychological well-being.

The difference in the results of mediation analysis across both the studies (Study 1 and Study 2) could be explained either due to the difference in the sample size or due to the difference in the timings (one month in Study 1 and 1 week in Study 2) of psychometric assessment conduction. However, the reasons for the differences in the results are complicated to understand because the participants in both studies were from different sports including team and individual sports, playing at different competitive levels, and belonging to different demographic backgrounds. Nonetheless, our study provides preliminary evidence that AME is a protective factor in preserving athletes’ PWB even after an unsatisfactory competitive encounter. It is to be noted that Athletic mental energy can also be useful in a sport player’s day-to-day life through the transfer effects. Gould and Carson^83^ suggested that athletes learn intrapersonal, interpersonal, cognitive, and behavioral skills from sports and adapt them to their daily lives^83^. Thus, athletes with high AME will not just benefit in sports but will also benefit in handling stressors from daily life. However, this is only one possibility we can make through our study. Future studies are advised to examine how AME helps athletes deal with adversities in daily life. In addition to this, our study provides several other implications for the researchers.

Our study highlights the vital role of a protective factor in sports excellence, athletic success, and well-being^33–35,44,65,70^. Despite proposals in the 1990s about mental energy’s impact on player performance, empirical reports remain limited. Our research addresses this gap by exploring the affective and cognitive components of mental energy and their connections to athlete performance and psychological well-being, complementing Lu et al.^79^. We build on Chuang, Lu, Gill, and Fang’s work^38^ by examining the interplay between athletes’ performance, mental energy, and psychological states, suggesting the need for post-competition mental state management^5,19,21,34^.

Lastly, athletes from joint families, nuclear families, and single-parent families showed distinct differences in psychological well-being (PWB) mean scores, highlighting variations across family structures. This aligns with Ryan and Willits^84^, emphasizing the impact of family relationships on well-being^84,85^. However, no significant differences emerged in Athletic Mental Energy (AME) among athletes based on sex or family structure. Similarly, PWB showed no significant variation between male and female athletes, indicating that neither sex nor family structure influences AME or PWB. In conclusion, our findings suggest that sex and family structure do not affect players’ psychological well-being or athletic mental energy. Therefore, enhancing AME through interventions may assist athletes in managing emotions and stress associated with suboptimal performance while promoting well-being and preparing them for future competitions. However, it is essential to note that additional research is necessary to corroborate our primary findings.

### Practical applications

Athletic Mental Energy (AME) holds a central role in sports psychology, predicting success, reducing athlete stress and burnout, and enhancing positive mental states^13,31,48^. Our study emphasizes AME’s significance, showing it as a mediator between athlete performance and psychological well-being. Sports professionals can use this insight in athlete training, especially post-competition, to help manage performance-related stress and improve well-being^86^. Lu et al.^79^ suggest cultivating AME through mental and physical training, influenced by factors like nutrition, sleep, relationships, and time management. Sport psychologists can integrate psychological skills training into daily routines, while tailored plans for healthy habits, routines, and sleep cycles can optimize mental energy levels^31,48^.

### Strengths and limitations

Our research employed an empirical approach to delve into the mediating role of Athletic Mental Energy (AME) in the post-competitive performance-psychological well-being dynamic, encompassing two distinct studies with separate participant groups. Employing diverse assessment methods is a research strategy aimed at gaining a comprehensive understanding, and in this pursuit, we used two different assessment approaches^20,87^. However, it’s important to acknowledge the limitations of our study. Firstly, the cross-sectional nature of our research limits its ability to establish variable patterns over time. Future studies would benefit from adopting a longitudinal design to explore the intricate relationship between athletes’ performance, AME, and psychological well-being over extended periods, providing a more accurate depiction of temporal changes. We excluded parasport participants, warranting investigation in this context. Cultural variations may affect generalizability, necessitating exploration in different settings. While we employed subjective performance measures to accommodate the diversity of sports, employing objective performance metrics and expanding the sample size would enhance generalizability. Additionally, measuring AME closer to competition would yield more precise results^25,45,58,59,88^. Lastly, the evolving concept of AME requires further examination, identifying potential latent factors beyond the six psychological components explored and their relevance in athletes’ post-competition well-being.(Supplementary information)

## Conclusions

Our research examined the mediating role of AME in the link between post-competition performance and psychological well-being. We found that athletes’ performance significantly impacts their psychological well-being through factors within athletic mental energy, including confidence, motivation, concentration, vigor, tirelessness, and calmness. We recommend sports psychologists and professionals prioritize interventions that enhance athletes’ AME, especially after competitions. This equips athletes to effectively handle performance-related stress and improves their overall well-being. Additionally, we advocate for further research to explore the positive aspects and constituent elements of AME in sports, aiming to advance athlete welfare and performance.$\overline{{\text{X}}}$

### Supplementary Information

Supplementary Information.

Supplementary Tables.

## Data Availability

The data analysed for this research paper are available from the first and corresponding author upon fair request meeting institutional guidelines.

## Abbreviations

N: Sample size
AME: Athletic mental energy
AMES: Athletic mental energy scale
PWB: Psychological wellbeing
SP: Subjective performance
VIG: Vigor
CONF: Confidence
MOT: Motivation
CON: Concentration
TIR: Tireless
CAL: Calm
ILSI: International life science institute
SPSS: Statistical package for social sciences
APA: American psychological association
α: Level of statistical significance alpha
IZOF: Individual zone of optimal functioning
H: Items of AME
F: Items of PWB
AS: Amisha Singh
MKA: Mandeep Kaur Arora
BB: Bahniman Boruah
