# Supplementary material for: The role of the six factors model of athletic mental energy in mediating athletes’ well-being in competitive sports
Source: Sci Rep. 2024 Feb 5;14:2974. doi: 10.1038/s41598-024-53065-5 (PMC10844369; doi:10.1038/s41598-024-53065-5)
Supplement: Supplementary file 1 — Supplementary Information. [file 41598_2024_53065_MOESM1_ESM.docx]

Annexures

**Section A**

**Demographic Profile (Self-reported)**

Please provide your basic information below.

Nationality:

State of permanent residence:

District:

Circle only one option:

Male Female Third Gender

Circle only one option:

Person with disability Person without disability

Date of birth: ____________________________________________

What is your current age in years? ____________________________

Educational details (*Tick only completed ones*):

- - Matriculation (≤ Grade 10)
  - Intermediate (12^th^ PASS)
  - High school diploma or equivalent
  - Vocational training
  - Graduate (Bachelor’s degree)
  - Postgraduate (Master’s degree)
  - Other, please specify: __________________________________________

Current Education/occupation status (*Example: Pursuing graduation/ currently employed/ full-time sport performer*): ___________________________________________________

Family structure (*Circle only one option*):

Joint family Nuclear family Single-parent family

**Sports profile**

Currently involved in (*Circle only one option*):

Team sports Individual sports

Currently playing in (*Circle only one option*):

Junior category Senior category

Name the sport you're currently involved in:

__________________________________________________________________________

Total no. of years active in sports: ______________________________________________

Please specify the number of years of playing sport at competitive levels. (*Specify in years*) __________________________________________________________________________

Your attendance for the past month in sport training is (*Circle only one option*):

Regular training session Irregular training session

Highest level of competition you have participated in (*Circle only one option*):

Local competition Regional competition State competition

National competition International competition Professional level

**Section B**

**Questionnaires** **(Self-reported)**

Please circle one number below to indicate how satisfied you have been, on average, with your performances in sporting encounters over the past month/past week.

During my sporting encounters over the past month, my performance left me feeling...

| **Totally Dissatisfied** **Totally Satisfied**  0 1 2 3 4 5 6 7 8 9 10 |
| --- |

| Please tick (✔) your response in only one box for each statement to indicate how much you agree or disagree. | | | | | | | |
| --- | --- | --- | --- | --- | --- | --- | --- |
| *Statement* | Strongly  agree | Somewhat  agree | A little  agree | Neither agree nor disagree | A little disagree | Somewhat disagree | Strongly disagree |
| “I like most parts of my personality.” |  |  |  |  |  |  |  |
| “When I look at the story of my life, I am pleased with how things have turned out so far.” |  |  |  |  |  |  |  |
| “Some people wander aimlessly through life, but I am not one of them.” |  |  |  |  |  |  |  |
| “The demands of everyday life often get me down.” |  |  |  |  |  |  |  |
| “In many ways I feel disappointed about my achievements in life.” |  |  |  |  |  |  |  |
| “Maintaining close relationships has been difficult and frustrating for me.” |  |  |  |  |  |  |  |
| “I live life one day at a time and don't really think about the future.” |  |  |  |  |  |  |  |
| “In general, I feel I am in charge of the situation in which I live.” |  |  |  |  |  |  |  |
| “I am good at managing the responsibilities of daily life.” |  |  |  |  |  |  |  |
| “I sometimes feel as if I've done all there is to do in life.” |  |  |  |  |  |  |  |
| “For me, life has been a continuous process of learning, changing, and growth.” |  |  |  |  |  |  |  |
| “I think it is important to have new experiences that challenge how I think about myself and the world.” |  |  |  |  |  |  |  |
| “People would describe me as a giving person, willing to share my time with others.” |  |  |  |  |  |  |  |
| “I gave up trying to make big improvements or changes in my life a long time ago” |  |  |  |  |  |  |  |
| “I tend to be influenced by people with strong opinions” |  |  |  |  |  |  |  |
| “I have not experienced many warm and trusting relationships with others.” |  |  |  |  |  |  |  |
| “I have confidence in my own opinions, even if they are different from the way most other people think.” |  |  |  |  |  |  |  |
| “I judge myself by what I think is important, not by the values of what others think is important.” |  |  |  |  |  |  |  |

| Below are 18 statements that describe “**how did you feel over the past month/week**” in sports training/ competition. Please tick (✔) a box that mostly represents your feelings. | | | | | | |
| --- | --- | --- | --- | --- | --- | --- |
| *Statement* | Completely not | Hardly ever | A little bit | Much | Very much | Completely so |
| I feel spiritual to do everything in sports |  |  |  |  |  |  |
| I feel there is endless energy coming from my body |  |  |  |  |  |  |
| I feel I can win all the competitions in the future |  |  |  |  |  |  |
| I feel excited in future competitions |  |  |  |  |  |  |
| There’s nothing distracting me in competition |  |  |  |  |  |  |
| There’s nothing distracting me in training |  |  |  |  |  |  |
| No matter how long the training lasts I don’t feel tired |  |  |  |  |  |  |
| I am full of passion to attend my sports |  |  |  |  |  |  |
| I can have my sports movements and skills automatically executed in sports |  |  |  |  |  |  |
| I am free of distraction during competition and training |  |  |  |  |  |  |
| Even the competition is over I still feel I have endless energy to use |  |  |  |  |  |  |
| Even the training is over I still feel I have endless energy to use |  |  |  |  |  |  |
| I can control all sports movements and skills |  |  |  |  |  |  |
| When facing to my opponents I am calm |  |  |  |  |  |  |
| Either in competition or training, I feel full of energy |  |  |  |  |  |  |
| I want to show my best to others in sports |  |  |  |  |  |  |
| Facing coming competitions, I don’t feel anxious |  |  |  |  |  |  |
| Even facing a tough opponent, I don’t feel anxious |  |  |  |  |  |  |
