# Supplementary material for: The role of the six factors model of athletic mental energy in mediating athletes’ well-being in competitive sports
Source: Sci Rep. 2024 Feb 5;14:2974. doi: 10.1038/s41598-024-53065-5 (PMC10844369; doi:10.1038/s41598-024-53065-5)
Supplement: Supplementary file 2 — Supplementary Tables. [file 41598_2024_53065_MOESM2_ESM.docx]

**The Role of the Six Factors Model of Athletic Mental Energy in Mediating Athletes’ Well-being in Competitive Sports**

Amisha Singh^1*^, Mandeep Kaur Arora^2,^ & Bahniman Boruah^1^

^1^Department of Psychology, University of Delhi, India

^2^ Kamala Nehru College, Department of Psychology, University of Delhi, India

**Author Note**

Amisha Singh
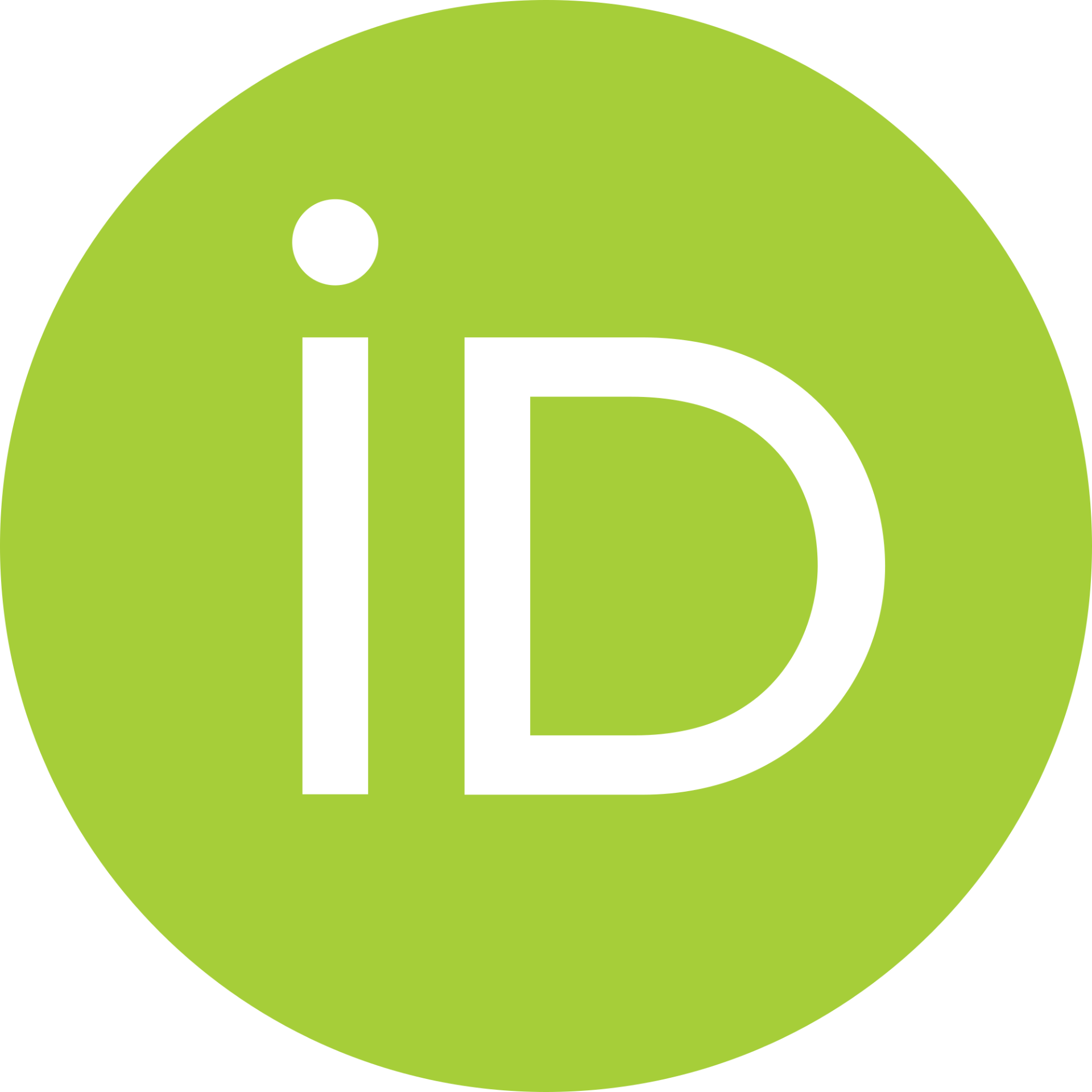
 <https://orcid.org/0000-0003-4456-3510>

*Correspondence concerning this research paper should be addressed to Amisha Singh, University of Delhi, Delhi 110007, India.

Email: [samisha987@gmail.com](mailto:asingh1@psychology.du.ac.in)

Table 1. Cronbach-Alpha of the scale (n=50)

*Reliability Statistics*

| Constructs | No. of Items | Alpha (α) |
| --- | --- | --- |
| AMES | 18 | .926 |
| PWB | 18 | .787 |

AMES, *Athletic Mental Energy Scale*; PWB, *Psychological Well-being*

Table 2.

*Frequency Distribution for Demographic and Sports Profile*

| Gender | Frequency | Percent | |
| --- | --- | --- | --- |
| Female | 25 | | 50 |
| Male | 25 | | 50 |
| Total | 50 | | 100.0 |
| State of Residence | Frequency | | Percent |
| Bihar | 1 | | 2 |
| Delhi | 25 | | 50 |
| Haryana | 7 | | 14 |
| Himachal Pradesh | 2 | | 4 |
| Jharkhand | 2 | | 4 |
| Madhya Pradesh | 1 | | 2 |
| Manipur | 1 | | 2 |
| Rajasthan | 3 | | 6 |
| Mumbai | 1 | | 2 |
| Uttar Pradesh | 7 | | 14 |
| Total | 50 | | 100.0 |
| Family Structure | Frequency | | Percent |
| Joint family | 17 | | 34 |
| Nuclear family | 19 | | 38 |
| Single-parent family | 14 | | 28 |
| Total | 50 | | 100.0 |
| Sport Category A | Frequency | | Percent |
| Individual sports | 19 | | 38 |
| Team sports | 31 | | 62 |
| Total | 50 | | 100.0 |
| Sport Category B | Frequency | | Percent |
| Junior category | 13 | | 26 |
| Senior category | 37 | | 74 |
| Total | 50 | | 100.0 |
| Sport | Frequency | | Percent |
| Archery | 6 | | 12 |
| Athletics | 7 | | 14 |
| Bodybuilding | 1 | | 2 |
| Baseball | 1 | | 2 |
| Boxing | 1 | | 2 |
| Kho-Kho | 1 | | 2 |
| Rock Climbing | 1 | | 2 |
| Table tennis | 1 | | 2 |
| Sprinting | 1 | | 2 |
| Taekwondo | 1 | | 2 |
| Cricket | 3 | | 6 |
| Hockey | 3 | | 6 |
| Football | 12 | | 24 |
| Kabaddi | 4 | | 8 |
| Volleyball | 7 | | 14 |
| Total | 50 | | 100.0 |
| Regularity in sports | Frequency | | Percent |
| Irregular training session | 15 | | 30.0 |
| Regular training session | 35 | | 70.0 |
| Total | 50 | | 100.0 |
| Sports level | Frequency | | Percent |
| National competition | 24 | | 48.0 |
| Professional level | 4 | | 8.0 |
| International level | 5 | | 10.0 |
| State competition | 14 | | 28.0 |
| Regional level | 2 | | 4.0 |
| Local level | 1 | | 2.0 |
| Total | 31 | | 100.0 |

Table 3. *Descriptive statistics of the factors of AME, PWB, and SP constructs*

| AME Factors | N | *X̄* | SD | Minimum | Maximum |
| --- | --- | --- | --- | --- | --- |
| Vigor | 50 | 4.2467 | 1.22855 | 1.33 | 6.00 |
| Confidence | 50 | 4.2400 | 1.03244 | 1.67 | 6.00 |
| Motivation | 50 | 4.6267 | 1.04900 | 2.33 | 6.00 |
| Concentration | 50 | 4.4133 | 1.05099 | 2.00 | 6.00 |
| Tireless | 50 | 4.0133 | 1.40933 | 1.00 | 6.00 |
| Calm | 50 | 3.8933 | 1.43244 | 1.00 | 6.00 |
| AME | 50 | 4.2389 | .97895 | 1.89 | 6.00 |
| PWB Factors | N | *X̄* | SD | Minimum | Maximum |
| Autonomy | 50 | 4.7333 | 1.09834 | 2.33 | 7.00 |
| Environmental Mastery | 50 | 4.8933 | .92445 | 2.33 | 6.67 |
| Personal Growth | 50 | 5.2800 | 1.17680 | 2.33 | 7.00 |
| Positive Relations with Others | 50 | 4.2733 | 1.05127 | 2.00 | 6.33 |
| Purpose in Life | 50 | 4.6133 | 1.09080 | 2.67 | 6.67 |
| Self-Acceptance | 50 | 5.1200 | .95181 | 3.33 | 7.00 |
| PWB | 50 | 4.8189 | .74737 | 3.39 | 6.44 |
| SP | 50 | 6.360 | 1.6630 | 4.0 | 10.0 |

*N* Sample Size; *AME* Athletic Mental Energy*; PWB* Psychological Wellbeing; *SP* Subjective Performance; *SD* Standard Deviation; *X̄* Mean

Table 4. *The overall mean score on Psychological Well-being across different family structures*

| Family structure | *X̄* | N | SD |
| --- | --- | --- | --- |
| Joint family | 83.667 | 15 | 21.9762 |
| Nuclear family | 83.500 | 16 | 20.0167 |
| Single-parent family | 83.684 | 19 | 16.3199 |
| Total | 83.620 | 50 | 18.9337 |

*N* Sample Size; *SD* Standard Deviation; *X̄* Mean

Table 5 *Mediation analysis representing the relationship between Sport performance and psychological well-being with athletic mental energy as a mediator.*

| Relationship | Total Effect | Direct Effect | Indirect Effect | Confidence Interval | | t-statistics | Conclusion |
| --- | --- | --- | --- | --- | --- | --- | --- |
|  |  |  |  | Lower Bound | Upper Bound |  |  |
| SP -> AME -> PWB | 10.8312  (p=0.000) | 9.5872  (p=0.000) | 1.2440 | -1.3081 | 3.5195 | 7.0412 | Insignificant mediation |

*SP* Subjective performance; *AME* Athletic Mental Energy; *PWB* Psychological well-being

Table 6. Cronbach-Alpha of AME and PWB scale (n=100)

*Reliability Statistics*

| Constructs | N | Alpha (α) |
| --- | --- | --- |
| AMES | 18 | .935 |
| PWB | 18 | .742 |

N, *No. of Items*; AMES, *Athletic Mental Energy Scale*; PWB, *Psychological Well-being*

Table 7.

*Frequency Distribution for Demographic and Sports Profile*

| Gender | | | Frequency | | | | Percent | | | | |  |
| --- | --- | --- | --- | --- | --- | --- | --- | --- | --- | --- | --- | --- |
| Female | | | 50 | | | | | 50 | | | |  |
| Male | | | 50 | | | | | 50 | | | |  |
| Total | | | 100 | | | | | 100.0 | | | |  |
| Permanent Residence | | | Frequency | | | | | Percent | | | |  |
|  | Arunachal Pradesh | | 1 | | | | | 1.0 | |  |  |  |
|  | Bhopal | | 1 | | | | | 1.0 | |  |  |  |
|  | Bihar | | 1 | | | | | 1.0 | |  |  |  |
|  | Chhattisgarh | | 1 | | | | | 1.0 | |  |  |  |
|  | Delhi | | 53 | | | | | 53.0 | |  |  |  |
|  | Haryana | | 18 | | | | | 18.0 | |  |  |  |
|  | Jammu & Kashmir | | 1 | | | | | 1.0 | |  |  |  |
|  | Jharkhand | | 1 | | | | | 1.0 | |  |  |  |
|  | Manipur | | 1 | | | | | 1.0 | |  |  |  |
|  | Mumbai | | 1 | | | | | 1.0 | |  |  |  |
|  | Punjab | | 1 | | | | | 1.0 | |  |  |  |
|  | Rajasthan | | 2 | | | | | 2.0 | |  |  |  |
|  | Uttar Pradesh | | 17 | | | | | 17.0 | |  |  |  |
|  | Uttarakhand | | 1 | | | | | 1.0 | |  |  |  |
|  | Total | | 100 | | | | | 100.0 | |  |  |  |
| Family Structure | | | | | | Frequency | | | | Percent | | |
| Joint family | | | | | 34 | | | | | 34 | | |
| Nuclear family | | | | | 34 | | | | | 34 | | |
| Single-parent family | | | | | 32 | | | | | 32 | | |
| Total | | | | | 100 | | | | | 100.0 | | |
| Sport Category Type 1 | | | | | Frequency | | | | | Percent | | |
| Individual sports | | | | | 22 | | | | | 22 | | |
| Team sports | | | | | 78 | | | | | 78 | | |
| Total | | | | | 100 | | | | | 100.0 | | |
| Sport Category Type 2 | | | | | Frequency | | | | | Percent | | |
| Junior category | | | | | 22 | | | | | 22 | | |
| Senior category | | | | | 78 | | | | | 78 | | |
| Total | | | | | 100 | | | | | 100.0 | | |
| Sport | | | | | Frequency | | | | | Percent | | |
| Aerobics | | | | | 3 | | | | | 3.0 | | |
| Archery | | | | | 1 | | | | | 1.0 | | |
| Athletics | | | | | 15 | | | | | 15.0 | | |
| Badminton | | | | | 4 | | | | | 4.0 | | |
| Baseball | | | | | 1 | | | | | 1.0 | | |
| Basketball | | | | | 11 | | | | | 11.0 | | |
| Cricket | | | | | 3 | | | | | 3.0 | | |
| Football | | | | | 20 | | | | | 20.0 | | |
| Handball | | | | | 2 | | | | | 2.0 | | |
| Hockey | | | | | 4 | | | | | 4.0 | | |
| Kho-kho | | | | | 8 | | | | | 8.0 | | |
| roller skating | | | | | 1 | | | | | 1.0 | | |
| Sprinting | | | | | 2 | | | | | 2.0 | | |
| Table Tennis | | | | | 2 | | | | | 2.0 | | |
| Volleyball | | | | | 23 | | | | | 23.0 | | |
| Total | | | | | 100 | | | | | 100.0 | | |
| Regularity in sport training | | | | | Frequency | | | | | Percent | | |
| Irregular training session | | | | | 22 | | | | | 22.0 | | |
| Regular training session | | | | | 78 | | | | | 78.0 | | |
| Total | | | | | 100 | | | | | 100.0 | | |
| Highest level of Competition Participated in | | | | | Frequency | | | | | Percent | | |
| International competition | | | | | 2 | | | | | 2.0 | | |
| National competition | | | | | 40 | | | | | 40.0 | | |
| Professional level | | | | | 3 | | | | | 3.0 | | |
| State competition | | | | | 18 | | | | | 18.0 | | |
| Regional level | | | | | 16 | | | | | 16.0 | | |
| Local level | | | | | 21 | | | | | 21.0 | | |
| Total | | | | | 100 | | | | | 100.0 | | |

Table 8. *Descriptive statistics of the factors of AME, PWB, and SP constructs*

| AME Factors | N | X̄ | SD | Minimum | Maximum |
| --- | --- | --- | --- | --- | --- |
| Vigor | 100 | 4.1767 | 1.13949 | 1.33 | 6.00 |
| Confidence | 100 | 4.0400 | 1.13735 | 1.33 | 6.00 |
| Motivation | 100 | 4.6133 | 1.10373 | 2.00 | 6.00 |
| Concentration | 100 | 4.1067 | 1.12504 | 1.33 | 6.00 |
| Tireless | 100 | 3.9800 | 1.19466 | 1.00 | 6.00 |
| Calm | 100 | 3.8567 | 1.19694 | 1.00 | 6.00 |
| AME | 100 | 4.1289 | .95473 | 2.00 | 5.83 |
| PWB Factors | N | X̄ | SD | Minimum | Maximum |
| Autonomy | 100 | 4.7600 | .95708 | 2.33 | 7.00 |
| Environmental Mastery | 100 | 4.8233 | .81161 | 2.33 | 6.67 |
| Personal Growth | 100 | 5.3200 | 1.08030 | 2.33 | 7.00 |
| Positive Relations with Others | 100 | 4.2067 | 1.07495 | 2.00 | 6.33 |
| Purpose in Life | 100 | 4.5900 | 1.07398 | 2.67 | 6.67 |
| Self-Acceptance | 100 | 5.0667 | .82878 | 3.33 | 7.00 |
| PWB | 100 | 4.7944 | .65442 | 3.39 | 6.44 |
| SP | 100 | 6.220 | 1.6489 | 4.0 | 10.0 |

*N* Sample Size; *AME* Athletic Mental Energy; *PWB* Psychological Wellbeing; *SP* Subjective Performance; *SD* Standard Deviation; *X̄* Mean

Table 9. *The mean score on the PWB across family structure*

| Family structure | *X̄* | N | SD |
| --- | --- | --- | --- |
| Joint family | 83.853 | 34 | 17.8464 |
| Nuclear family | 77.853 | 34 | 14.2789 |
| Single-parent family | 86.719 | 32 | 21.5260 |
| Total | 82.730 | 100 | 18.2474 |

*N* Sample Size; *SD* Standard Deviation; *X̄* Mean

Table 10. *Differences in Athletic Mental Energy and Psychological Wellbeing between Male and Female Sport Performers.*

|  | |  |  | Levene's Test for Equality of Variances | | t-test for Equality of Means | | | | | | |
| --- | --- | --- | --- | --- | --- | --- | --- | --- | --- | --- | --- | --- |
|  |  |  |  | F | Sig. | t | df | Sig. (2-tailed) | Mean Difference | Std. Error Difference | 95% Confidence Interval of the Difference | |
|  |  | Mean | SD |  |  |  |  |  |  |  | Lower | Upper |
| PWB TOT. | Male | 81.040 | 17.1714 | 1.412 | .238 | -.925 | 98 | .357 | -0.185 | 0.200 | -0.582 | .211 |
|  | Female | 84.420 | 19.2885 |  |  |  |  |  |  |  |  |  |
| AME TOT. | Male | 71.260 | 15.4071 | 1.195 | .277 | -.560 | 98 | .577 | -0.112 | 0.200 | -0.510 | 0.285 |
|  | Female | 73.160 | 18.4173 |  |  |  |  |  |  |  |  |  |

*M* Mean; *SD* Standard Deviation; *PWB* Psychological Well-being; *AME* Athletic Mental Energy

Table 11. *Athletic Mental Energy across joint, nuclear, and single-parent family*

|  |  |  | Test of Homogeneity of Variances | | ANNOVA | |
| --- | --- | --- | --- | --- | --- | --- |
| Family Structures | Mean | Std. Deviation |  | | F | Sig. |
|  |  |  | Levene’s Statistic | Sig. |  |  |
| Joint | 73.324 | 16.7508 |  |  | 1.445 | .241 |
| Nuclear | 68.353 | 15.5950 | .744 | .478 |  |  |
| Single-parent | 75.125 | 18.1726 |  |  |  |  |

Table 12. *Mediation analysis represents the relation between performance and psychological well-being with athletic mental energy as a mediator.*

| Relationship | Total Effect | Direct Effect | Indirect Effect | Confidence Interval | | t-statistics | Conclusion |
| --- | --- | --- | --- | --- | --- | --- | --- |
|  |  |  |  | Lower Bound | Upper Bound |  |  |
| SP-> AME -> PWB | 8.972  (p=0.000) | 6.998  (p=0.000) | 1.973 | 0.442 | 3.712 | 2.333 | Partial Mediation |

*SP* Subjective performance; *AME* Athletic Mental Energy; *PWB* Psychological well-being

Table 13. *Mediation analysis of 6 factors of AMES*

| Relationship | Total Effect | Direct Effect | Indirect Effect | Confidence Interval | | t-statistics | Conclusion |
| --- | --- | --- | --- | --- | --- | --- | --- |
|  |  |  |  | Lower Bound | Upper Bound |  |  |
| SP-> VIG -> PWB | 8.972  (p=0.000) | 7.204  (p=0.000) | -0.410 | 1.766 | 1.539 | 3.416 | Partial Mediation |
| SP-> CONF -> PWB | 8.972  (p=0.000) | 7.204  (p=0.000) | 0.513 | 0.6434 | 1.870 | 2.331 | Partial Mediation |
| SP-> MOT -> PWB | 8.972  (p=0.000) | 7.204  (p=0.000) | 0.647 | 0.310 | 1.687 | 2.776 | Partial Mediation |
| SP-> CON -> PWB | 8.972  (p=0.000) | 7.204  (p=0.000) | 0.002 | 0.888 | 1.139 | 2.222 | Partial Mediation |
| SP-> TIR -> PWB | 8.972  (p=0.000) | 7.204  (p=0.000) | 0.687 | 0.937 | 2.806 | 1.962 | Partial Mediation |
| SP-> CAL -> PWB | 8.972  (p=0.000) | 7.204  (p=0.000) | 0.327 | 0.158 | 0.936 | 2.165 | Partial Mediation |

*SP* Subjective performance; *VIG* Vigor; *CONF* Confidence; *MOT* Motivation; CON Concentration; *TIR* Tireless; *CAL* Calm; *PWB* Psychological well-being
